# Supplementary material for: Reproductive Potential and Population Growth of the Worm Enchytraeus buchholzi (Clitellata: Enchytraeidae) Under Laboratory Conditions as Well as Regression Models
Source: Biology (Basel). 2025 Feb 6;14(2):167. doi: 10.3390/biology14020167 (PMC11851633; doi:10.3390/biology14020167)
Supplement: Supplementary file 1 [file biology-14-00167-s001.zip › biology-3412331-supplementary.pdf]

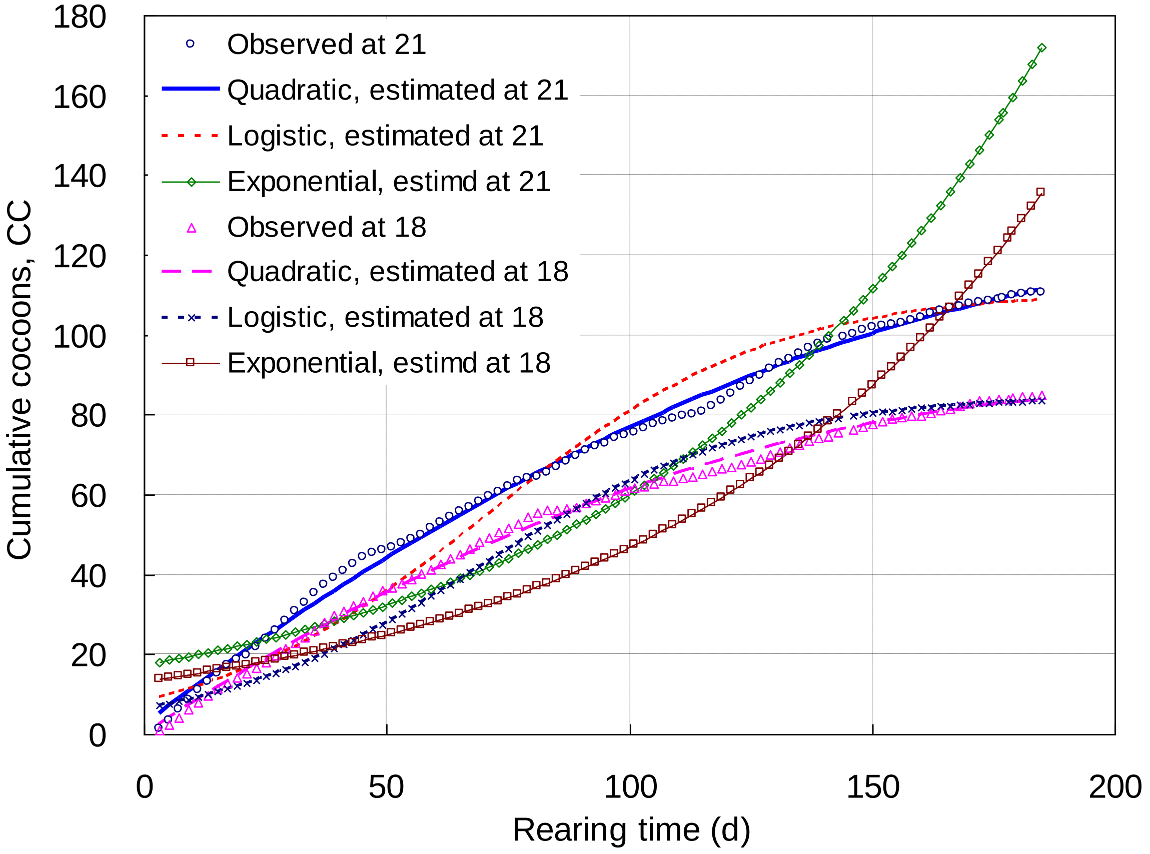

Figure S1. Integral curves of cumulative cocoons (*CC*) laid by an adult of *Enchytraeus buchholzi* reared at 18 or 21 °C respectively, with the tracks of the real exponential equations shown.

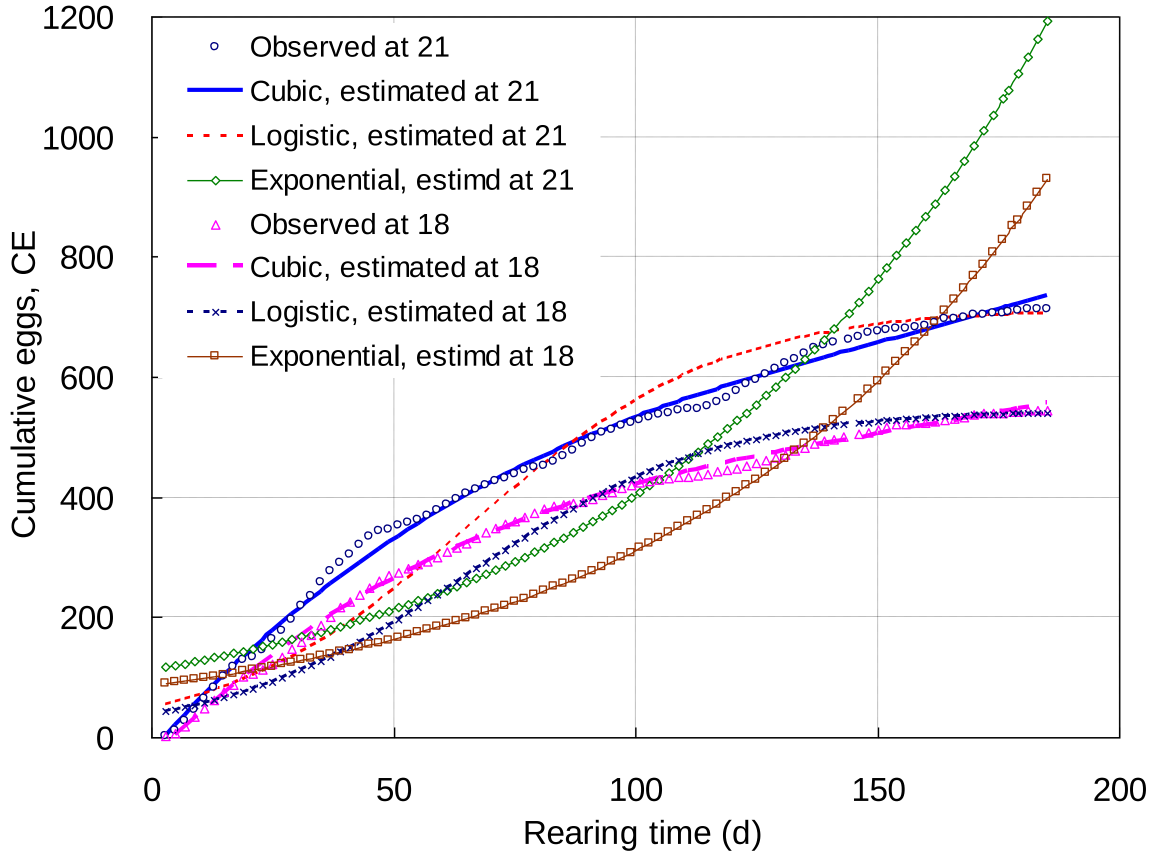

Figure S2. Integral curves of cumulative eggs (*CE*) laid by an adult of *Enchytraeus buchholzi* reared at 18 or 21 °C respectively, with the tracks of the real exponential equations shown.
